# Effectiveness of an educational intervention to improve the safety culture in primary care: a randomized trial

**DOI:** 10.1186/s12875-018-0901-8

**Published:** 2019-01-18

**Authors:** Clara González-Formoso, Ana Clavería, M.J. Fernández-Domínguez, F.L. Lago-Deibe, Luis Hermida-Rial, Antonio Rial, Francisco Gude-Sampedro, Salvador Pita-Fernández, Victoria Martín-Miguel

**Affiliations:** 1 Vigo Teaching Unit of Family and Community Medicine and Nursing, EOXI Vigo, Galician Health Service, RedIAPP, Grupo I-Saúde (Instituto de Investigación Sanitaria Galicia Sur), Vigo, Spain; 2 Quality and Research Unit, Primary Care, EOXI Vigo, Galician Health Service, RedIAPP, Grupo I-Saúde (Instituto de Investigación Sanitaria Galicia Sur), Rosalía de Castro 21-23, 36201 Vigo, Spain; 3 Ourense Health Center, EOXI Ourense, Galician Health Service, RedIAPP, Grupo I-Saúde (Instituto de Investigación Sanitaria Galicia Sur), Ourense, Spain; 4 Sárdoma Health Center, EOXI Vigo, Galician Health Service, RedIAPP, Grupo I-Saúde (Instituto de Investigación Sanitaria Galicia Sur), Vigo, Spain; 5 Fontenla-Maristany Health Center, EOXI Ferrol, Galician Health Service, Ferrol, Spain; 6 Universidad de Santiago de Compostela, Santiago de Compostela, Spain; 7 Epidemiology Department, EOXI Santiago de Compostela, Instituto de Investigación Sanitaria Santiago de Compostela, Santiago de Compostela, Spain; 8 Clinical Epidemiology and Biostatistics Unit, EOXI A Coruña, A Coruña, Spain

**Keywords:** “Patient safety”[mesh], “Education”[mesh], “Health Services Research”[mesh], “Evaluation Studies as Topic”[mesh]

## Abstract

**Background:**

Fostering a culture of safety is an essential step in ensuring patient safety and quality in primary care. We aimed to evaluate the effectiveness of an educational intervention to improve the safety culture in the family and community medicine teaching units in an Atlantic European Region.

**Methods:**

Randomized study conducted in family and community medicine teaching units in Galicia (Spain). Participants were all fourth-year residents and their tutors (*N* = 138). Those who agreed to participate were randomized into one of two groups (27 tutors/26 residents in the intervention group, 23 tutors/ 23 residents in the control one).All were sent the Survey on Patient Safety Culture. After that, the intervention group received specific training in safety; they also recorded incidents over 15 days, documented them following a structured approach, and had feedback on their performance. The control group did not receive any action. All participants completed the same survey four months later. Outcome measures were the changes in safety culture as quantified by the results variables of the Survey: *Patient Safety Grade* and *Number of events reported.*

We conducted bivariate and adjusted analyses for the outcome measures. To explore the influence of participants’ demographic characteristics and their evaluation of the 12 dimensions of the safety culture, we fitted a multivariate model for each outcome.

**Results:**

Trial followed published protocol. There were 19 drop outs. The groups were comparable in outcome and independent variables at start. The experiment did not have any effect on *Patient safety grade* (− 0.040) in bivariate analysis. The odds of reporting one to two events increased by 1.14 (0.39–3.35), and by 13.75 (2.41–354.37) the odds of reporting 3 or more events. Different dimensions had significant independent effects on each outcome variable.

**Conclusion:**

A educational intervention in family and community medicine teaching units may improve the incidents reported. The associations observed among organizational dimensions and outcomes evidence the complexity of patient safety culture measurement and, also, show the paths for improvement. In the future, it would be worthwhile to replicate this study in teaching units from different settings and with different health professionals engaged.

**Trial registration:**

It was retrospectively registered with (ISRCTN41911128, 31/12/2010).

**Electronic supplementary material:**

The online version of this article (10.1186/s12875-018-0901-8) contains supplementary material, which is available to authorized users.

## Background

To prevent healthcare errors, it is essential to improve the safety culture [1, 2] because this underlies behaviors that ensure safety such as reporting and preventing incidents and adverse events [3, 4]. Organizational culture is a complex phenomenon that can be understood from different epistemological viewpoints, using different methods and assessment instruments [5] and there is a complex two-way relationship between safety culture and patient and staff outcomes [6]. In this context, it is essential to implement interventions to expand the safety culture [7] and to carry out studies to evaluate these interventions. However, rather than focusing on a particular procedure or technology, this process should focus on changing behavior through promoting leadership and teamwork.

In primary health care, fostering the safety culture is essential to guarantee patient safety in the future [8, 9] and is considered critical in continuing education and effective teamwork. Healthcare professionals, both tutors and residents, should be encouraged to identify safety issues and propose solutions to ensure patients’ safety. The role of tutors goes beyond supervision: tutors contribute positively or negatively as models of behavior for residents to emulate. The training and education of the former is as important as that to junior doctors. And the latter should have access to the most updated knowledge during specialization [10]. The method and techniques of effective training in skills acquisition for the qualification of professionals on patient safety are not trivial and has generated great interest in the scientific field [11, 12]. Not all intervention always gets the expected effect [13, 14].

Thus, we aimed to evaluate the effectiveness of an educational intervention to improve the safety culture in the family and community health teaching units of an Atlantic European Region.

## Methods

### Design

Randomized single-factor experimental study with two groups (intervention and control), from October 2008 to September 2009. The study protocol was published in BMC Family Practice [15].

### Setting and participants

The Spanish National Health System is a system of universal coverage with the territorial organization of health services based on health areas. In Galicia, with a population of 2.7 million inhabitants, there are 7 health areas, 398 primary care centers and 3141 family doctors, with an average of 1500 citizens. Each area has a teaching unit of Family and Community Medicine [16].

**Fig. 1:**
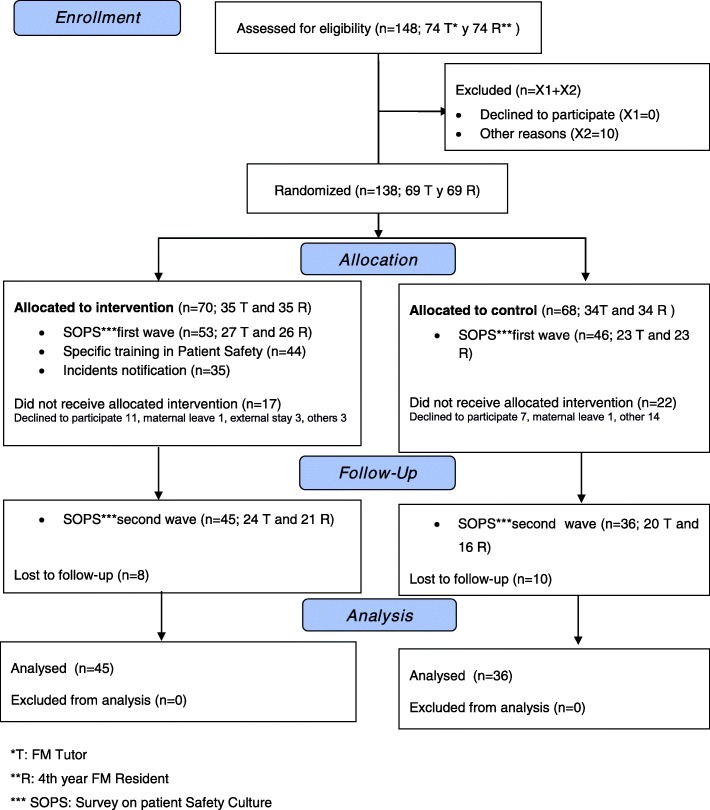
CONSORT 2010 Flow Diagram

Twenty-four tutor-resident units in each group are required to detect an improvement in patient safety culture as measured by Patient Safety Grade of 30%, with 80% power and confidence level of 95%. If we estimate 10% in losses, 27 tutor-resident units per group must be captured. Although, the project was also an opportunity for training improvement and all residents in their final year of training in the four-year residency program for the specialty of Family and Community Medicine and their tutors (69 pairs) were invited. Numbers of subjects and tutor-resident pairs that were assigned to the intervention and control groups respectively, as well as their follow-up, are detailed in Fig. 1.

### Intervention

We followed the published protocol [15]. Among those who agreed to participate, as stated in a written informed consent, each tutor-resident pair was randomly assigned to the intervention or control group through use of the SPSS 17, by one team researcher not belonging to Primary Care. The pair was assigned to groups by stratifying the teaching unit to ensure their equivalence in relation to a number of variables of interest and to avoid possible underlying biases. After randomization, we mailed the Survey On Patient Safety Culture (SOPS) directly to each physician; we also mailed three reminders requesting physicians to complete the survey.

Intervention consists of three different components:

- *Training workshops:* One training workshop was conducted in each of the 7 areas, given by one nurse and one family doctor together and lasted 2 h. Each participant was provided with current data on the incidence of adverse events in primary care and they were acquainted with current initiatives both in Spain and internationally. There was an introduction to patient safety: concepts of adverse effect, incident, adverse event, complication, secondary effect, adverse drug reaction; types of errors and their analysis; errors related to the use of drugs. The functioning of the form for reporting incidents was exercised.
- *Recording incidents* (the same days for all participants), using the methods employed in a large study of adverse events in primary care carried out by the Spanish Ministry of Health were each participant reported the incidents observed and data related to his or her daily activity over 15 days following APEAS form [15, 17].
- *Feedback:* At the end, each intervention participant received by email a report analyzing his or her registry.

Four months after the initial survey, all participants completed the SOPS again (Fig. 1).

### Measures

The SOPS [18] measures staff perceptions of patient safety culture in their work area/unit, as well as perceptions about patient safety culture in the organization as a whole. The following 12 dimensions of patient safety culture are included, with each dimension measured by 3 or 4 items: Communication openness, Feedback & communication about error, Frequency of events reported, Hospital handoffs & transitions, Hospital management support for patient safety, Nonpunitive response to error, Organizational learning-continuous improvement, Overall perceptions of safety, Staffing, Supervisor/manager expectations & actions promoting patient safety, Teamwork across hospital units, Teamwork within units. Dimensions, items and scores are described in Additional file 1. User’s Guide and other toolkit materials are available on the AHRQ Web site [18].

The survey also includes two outcome questions that ask respondents to provide an overall grade on patient safety for their work area/unit (A-Excellent scored as 1, B-Very Good scored as 2, C-Acceptable scored as 3, D-Poor scored as 4, E-Failing scoresd as 5) and to indicate the number of events they have reported over the past 12 months (No events, 1 to 2 events, 3 to 5 events, 6 to 10 events, 11 to 20 events, or 21 events or more). These were the dependent variables in our study (*Patient Safety Grade* and *Number of events reported*).

In addition, respondents were asked to provide limited background and demographic information about them.

The psychometric properties of the survey for its use in primary care were adequate, with Cronbach’s alpha between 0.60 and 0.95 for the dimensions of the SOPS [19].

To facilitate the analysis and reproducibility of the intervention, we used the Template for Intervention Description and Replication provided by the EQUATOR Network [20] (Additional file 2) and CONSORT [21].

### Statistical analysis

Following the recommendations of the Agency for Healthcare Research and Quality, we reverse coded negatively worded questions for the analysis. For each dimension, we calculated the mean score for each participant from the items making up the dimension. The variable *Number of events reported* was aggregated from 6 to 3 categories, due to the small number in more than 3 events reported (only one).

The outcomes of the experiment were *Patient Safety Grade,* a quantitative non parametric variable, and *Number of events reported,* qualitative with three categories.

To deal with missing data, we used SPSS version 19 to apply Little’s missing completely at random (MCAR) test. We compared those who completed the trial and those who dropped out by logistic regression including outcome, dimensions, age and sex in the model. To check the normality of the data distribution, we apply the Shapiro-Wilk test, because this test is best for small samples [22]. In the basal bivariate analysis, we used Mann-Whitney, Chi-square and Kruskall-Wallis tests as appropriate.

We used per protocol and intention-to-treat analysis to compare results between groups with several R’s packages [23, 24]. We applied *coin* [25] and *epitools* [26] to calculate the effect size with completed cases. For the quantitative outcome variable, the Wilcoxon statistic is divided by the square root of the sample size; for the qualitative with three categories, the odds ratio for each category is calculated by comparing it with the baseline.

We performed a multivariate imputation by chained equations, imputed the missing data 20 times, resulting in 5 completed data sets with *mice* [27] and made visual checks. We inspected the effect size in the five imputed sets. The results of the regression models were pooled.

To fit regression models with *Patient Safety Grade*, we first diagnosed its distribution as Gaussian [28] and selected generalized linear models [29, 30]. For *Number of events reported*, we used a multinomial model where the category “no event reports” and category “control group” were used as a reference. In both multivariate calculations, the 12 dimensions were included in the regression models as predictors and were adjusted by age, sex, and basal outcome value. We used the stepwise method to eliminate the independent variables without any statistically significant effect, applying the Akaike Information Criterion (AIC), followed by anova test. Unstandardized regression coefficients were used to express the B coefficients of the regression analyses. To check the appropriateness of the models, we visually inspected the residual plots.

Statistical significance was assessed at a level of < 0.05 (two-tailed).

## Results

The flow diagram in Fig. 1 shows the recruitment, randomization, assignment, intervention, and follow-up, with the participation in each phase. Of 138 participants at trial entry, 58.7% completed the trial. During the registration period, 140 reports were registered and there were 9045 practice visits. Prevalence was 1.3% for incidents (*n* = 82), 0.4% for adverse events (*n* = 35) with 23 notifications discarded because they were not related to health care.

In Table 1, we show the basal and post intervention descriptive analysis of the intervention and control groups, including sociodemographic characteristics and the 12 SOPS dimensions. As the quantitative variables were not normally distributed, we used medians and interquartile ranges to describe them.

**Table 1:** Independent variables (sociodemographic and 12 patient safety culture dimensions) characteristics by group, baseline and post intervention

	Intervention						Control					
	At baseline			At follow up			At baseline			At follow up		
	n (%)						n (%)					
Sex												
Man	27 (50.90)						19	(41.30)				
Women	26 (49.10)						27	(58.70)				
	n	median	(IQR)				n	median	(IQR)			
Age	53	44.00	(19.00)				46	36.50	(22.00)			
	n	median	(IQR)	n	median	(IQR)	n	median	(IQR)	n	median	(IQR)
SOPS dimensions												
D1. Teamwork Within Units	53	3.50	(1.00)	45	3.50	(0.75)	46	3.50	(0.75)	35	3.50	(0.75)
D2. Supervisor/Manager Expectations & Actions Promoting Patient Safety	53	3.75	(1.25)	45	4.00	(0.75)	46	3.75	(0.75)	36	3.63	(0.75)
D3. Organizational Learning—Continuous Improvement	53	2.67	(1.00)	43	2.67	(1.00)	46	3.00	(1.00)	36	3.17	(1.00)
D4. Management Support for Patient Safety	53	3.00	(1.00)	43	3.00	(1.33)	46	3.33	(1.00)	36	3.33	(1.17)
D5. Overall Perceptions of Patient Safety	53	3.25	(0.75)	44	3.00	(1.00)	46	3.25	(0.50)	36	3.25	(0.63)
D6. Feedback & Communication About Error	53	2.67	(0.67)	44	2.67	(1.00)	46	3.00	(1.33)	36	3.00	(1.00)
D7. Communication Openness	53	3.33	(1.00)	45	3.67	(1.00)	46	3.67	(0.67)	36	3.83	(0.67)
D8. Frequency of Events Reported	53	2.33	(1.33)	43	2.67	(1.00)	46	2.67	(1.67)	36	3.00	(1.00)
D9. Teamwork Across Units	53	3.50	(0.75)	41	3.00	(0.25)	46	3.38	(0.75)	36	3.00	(0.50)
D10. Staffing	53	2.75	(1.00)	45	3.00	(0.75)	46	3.00	(0.75)	36	3.25	(0.75)
D11. Handoffs & Transitions	53	3.25	(0.75)	39	3.25	(1.00)	46	3.38	(0.50)	35	3.25	(0.75)
D12. Nonpunitive Response to Error	53	3.00	(1.00)	45	3.00	(0.67)	46	3.33	(0.67)	36	3.00	(0.50)

No differences were observed between groups at start for any of the sociodemographic variables, dimensions or outcome variables (Tables 1 and 2). Other organizational characteristics are shown in Additional file 3.

**Table 2:** Summary results of the intervention for each outcome variable by group

	Intervention						Control						
	At baseline			At follow up			At baseline			At follow up			Effect size
	n	median	(IQR)	n	median	(IQR)	n	median	(IQR)	n	median	(IQR)	median
Patient safety grade	52	3.00	(1.00)	44	3.00	(1.00)	45	3.00	(0.00)	36	3.00	(0.00)	−0.04
	n (%)			n (%)			n (%)			n (%)			odds ratio (confidence interval)
N. events reported													
None	38	(74.51)		21	(46.67)		35	(76.09)		24	(68.57)		
1 to 2	12	(23.53)		10	(22.22)		10	(21.74)		10	(28.57)		1.14 (0.39–3.35)
3 or more	1	(1.96)		14	(31.11)		1	(2.17)		1	(2.86)		13.75 (2.41–354.37)

In the initial SOPS, losses were 2.0% for two items. In the final SOPS, the losses for the two outcome variables and the 12 dimensions were 20%, due to lost participants, as complete record missingness. The differences between completed cases and drop outs were not significant except for the dimension *Overall Perceptions of Patient Safety.* Little’s MCAR test showed that missing data were not MCAR and imputation was performed under missing at random assumption.

In the bivariate analysis (post-intervention groups), considering the outcome variable *Patient safety grade*, we detected that the effect of the intervention is small and non-significant, and it is presented as median difference (Table 2). As for the outcome *Number of events reported,* the odds of reporting one to two events increased by 1.14 (0.39–3.35) and the odds of reporting 3 or more events by 13.75 (2.41–354.37). The analysis was repeated with the five imputed sets, and inspection of the results confirm the previous one (data not shown).

The adjusted analysis with imputed data (Additional file 4) showed that group and initial outcome value did not have a positive effect on *Patient Safety Grade.* However, these variables did have an independent effect on *Number of events reported*, also adjusting for basal values, in the category “3 or more events reported”.

As exploratory analysis, we identified different SOPS dimensions which had an independent effect on each outcome, comparing intention-to-treat and per protocol regression models (Table 3).

**Table 3:** Summary results of the intervention for each outcome variable by group^a^

	Intention-to-treat				Per protocol			
Patient safety grade. Generalized lineal model, gaussian (identity link).								
Intercept	6.08***	(0.49)			6.27***	(0.48)		
Intervention group (control group = ref)	−0.19•	(0.11)			−0.26***	(0.12)		
D4. Management Support for Patient Safety	−0.67***	(0.10)			−0.66*	(0.11)		
D10. Staffing	−0.22*	(0.11)			−0.32*	(0.12)		
D2. Supervisor/Manager Expectations & Actions Promoting Patient Safety	0.19	(0.11)			0.23*	(0.11)		
D5. Overall Perceptions of Patient Safety	−0.30**	(0.12)			−0.32*	(0.14)		
Number of events reported. Multinomial model.								
	1 to 2 (none = ref)		3 or more (none = ref)		1 to 2 (none = ref)		3 or more (none = ref)	
Intercept	−2.78*	(1.09)	−3.47*	(1.45)	−3.05*	(1.30)	−3.74*	(1.63)
Intervention group (control group = ref)	0.20	(0.53)	2.69**	(1.02)	0.57	(0.62)	3.18**	(1.10)
D6. Feedback & Communication About Error	0.64•	(0.34)	0.18	(0.39)	0.72	(0.41)	0.21	(0.44)

^a^Data as Estimate (SD)

*** *p* < 0.001, ** *p* < 0.01, * *p* < 0.05, • < 0.1

**Fig. 2:**
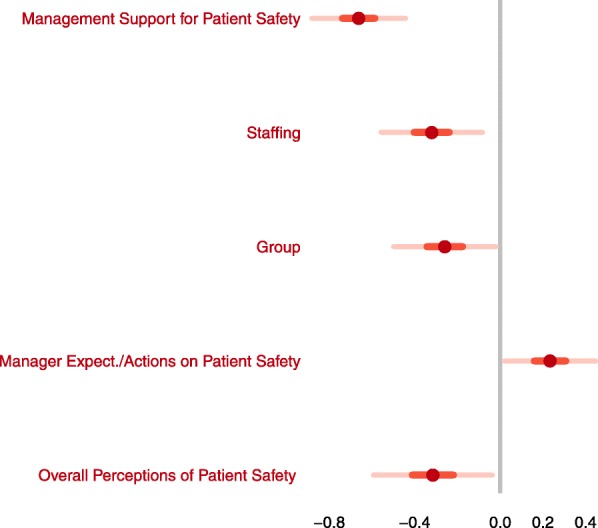
Generalized Lineal Model (outcome: Patient Safety Grade). Adjusted independent effects per protocol.Significant estimates are colored in red. Bars denote CIs

**Fig. 3:**
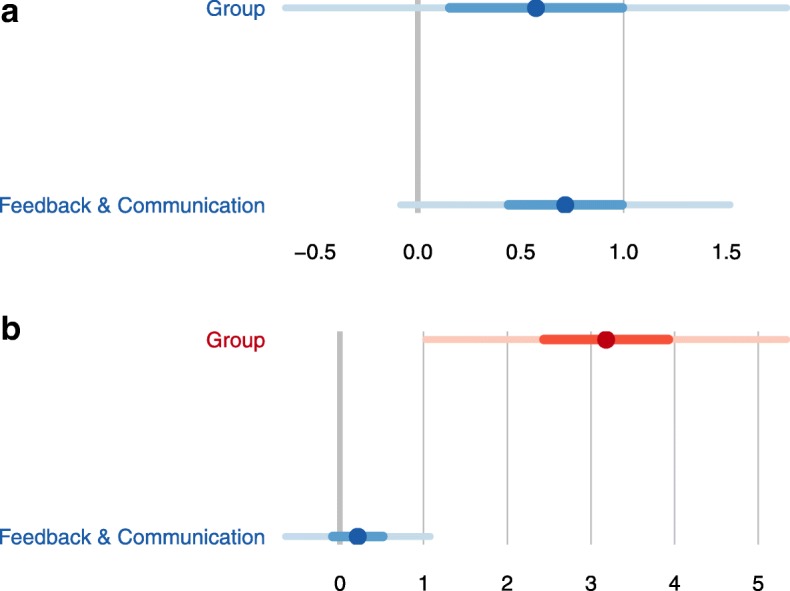
Multinomial model (outcome: Number of events reported). Adjusted independent effects per protocol. Panel A. Number of events reported 1–2. Significant estimates are colored in red. Bars denote CIs. Panel B. Number of events reported ≥3. Significant estimates are colored in red. Bars denote CIs

The dimensions *Management Support for Patient Safety, Staffing, Supervisor/Manager Expectations & Actions Promoting Patient Safety, Overall Perceptions of Patient Safety* had a significant effect on *Patient Safety Grade* and group reached almost significance*. O*n *Number of events reported,* group on “3 or more” had an adjusted independent effect on the category, and *Feedback & Communication about Error* was not significant in the category “1 to 2” but had a clear positive trend. The coefficients per protocol were similar, although, group had adjusted effect on *Patient Safety Grade* (Fig. 2) while *Feedback* did not have it on *Number of events reported* (Fig. 3). With basal data, only the dimension *Overall perception of Patient Safety* had an independent significant effect in both outcomes (data not shown).

## Discussion

### Effect of the intervention

We conducted a randomized single-factor experimental study with two groups (intervention and control), to evaluate the effectiveness of an educational intervention to change patient safety culture as measured by SOPS. Given the simplicity of the intervention, its effectiveness and large effect on *Number of events reported* is especially noteworthy, because the odds of reporting one to two events increased by 1.14 (0.39–3.35), which is a small effect, while the odds of reporting 3 or more events is 13.75 (2.41–354.37), a large one. The intervention consisted only of a two-hour workshop, the registration and analysis of incidents over 15 days and feedback. There were no differences in the *Patient Safety Grade* among the trial groups.

The use of the number of events reported as a measure of the safety culture has been the subject of debate [31]. On the one hand, a high number of reported events can indicate a large number of errors; on the other, it can indicate a robust safety culture that promotes the reporting of events without the fear that doing so will be held against staff [32]. Importantly, the dimension *Feedback and communication about error* also had a small positive effect on the *Number of events reported*. Thus, it is not only how many events occur or why they occur that is important, rather the reporting of events is an important component of organizational learning based on the flow of information. This dimension may also have been influenced by the fact that feedback was a component of the intervention.

Our study underlines the importance of dimensions related to organization, such as the role of leaders and staffing. *Patient Safety Grade*, increased with dimensions highly related to the health system macro organization, such as *Management Support for Patient Safety* and *Staffing,* and decreases with *Supervisor/Manager Expectations & Actions Promoting Patient Safety.* The study period was short and there were no mentionable changes made in the system. This may justify the fact that the differences by group in this outcome of the trial were very small.

Some authors would also consider the survey a component of the intervention [33, 34]. This interpretation is reasonable, under this assumption, we did not find any differences in the control group alone (pre and post intervention).

### Strengths and limitations

Although there are many papers on how to increase adverse events notification, to our knowledge, there are only two clinical trials that assessed the effectiveness of an intervention to improve the safety culture in primary care; both [34, 35] used different instruments to measure safety, both measured the effects of the intervention after one year, and started later than ours; only one found significant differences. Verbakel et al. [34] showed that practices involved in the workshop reported 42 (95% confidence interval [CI] = 9.81 to 177.50) times more incidents compared to the control group. The number of incidents was reported as quantitative variable and their basal data was much higher than in our organization. They did not find differences between groups at follow-up in Patient Safety Culture.

A systematic review on educational interventions [10] to improve patient safety for physicians in training selected 26 studies, but only one of these was a low quality clinical trial [36]. It supports the opportunity of studies such as ours. The participation of all seven training units in Galicia was a strong point of the intervention. But GPs and residents participated collaboratively in the project and, subsequently, some tutor-resident pairs responded together to the survey. This possibility was not anticipated by the research team and so, we cannot reliably adjust the results by this variable.

On the other hand, only 59% of the fourth-year residents invited to participate actually finished the study. Residents in the specialty of family and community medicine spend six to eight months in the primary care center during their fourth and final year, and some were lost to the second survey. No differences were found for dependent and independent variables among those who participated and those who did not. Using fourth-year residents as the subjects of the study also made it impossible to extend the follow-up period, as would probably be recommendable. For this reason, the effective time of incidents notification after the intervention was 4 months instead of 12, as it should be; but this circumstance acts against the difference between the results of both groups.

Under missing at random assumption, multiple imputation analyses will avoid bias only if enough variables predictive of missing values are included in the imputation model. To select the predictors, we followed Steve van Buuren procedures [24]. When complete-case analysis and multiple imputation analysis are compared, they are not identical but their variations are consistent between the two approaches. The estimates of standard errors under the multiple imputation are predominantly smaller, leading to narrower confidence intervals than under the complete case analysis.

Another drawback is that the instrument we used was designed for use in hospitals. Thus, some items, such as “We use more agency/temporary staff than is best for patient care”, were difficult to interpret, and this may have influenced our results. However, this was the only validated Spanish version available at the time of the study [37].

To minimize the risk of selection bias, the study was stratified by teaching units, and professionals who agreed to participate were informed of their assignment through a personal letter. To reduce information bias, we used a validated questionnaire [37, 38] and the same professionals conducted the training sessions. It is possible that some participants in the intervention group might have been averse to recording incidents and adverse events (unacceptability bias); however, the fact that participants knew that the questionnaires would be collected by third parties and anonymized favored truthfulness. To avoid contamination in the control group, we did not specify the objectives or hypotheses of the study. Although we cannot rule out a positive influence of the study in the safety culture of the control subjects; if such contamination did occur, it would decrease the differences between groups. To control for confounding effects between independent variables, we adjusted the regression models being aware of the non-parametric distribution of the variables and our small sample size. The independent variables and especially the dimensions of patient safety culture are interrelated in such a way that their different effects cannot be interpreted separately with any meaning. Moreover, we were interested in identifying what dimensions and to what extend they could influence the overall assessment of culture, as they point out aspects to be prioritized in a process of improvement.

We are also aware of the limitations of a quantitative approach to measuring safety culture, since some important components of safety culture can remain occult [39]. Focus groups, interviews, or direct observation of staff and patients could provide better information about some important aspects related to culture change [30]. Besides, socially desirable responding is a special concern when measuring individual differences with self-reports; to prevent this, the survey was sent individually by a team researcher outside the training units, and the feedback about declared incidents was also personalized.

### Importance of the topic

Education is a key pillar of quality improvement [40] and is considered the most important factor in improving patient safety in primary care [30, 41]. Growing evidence shows that training in patient safety improves knowledge and the process of care [42, 43], resulting in the proliferation of study plans and interventions that include education and training in patient safety [12, 44]. Nevertheless, few studies have involved senior physicians [45, 46], who play a key role in training residents. This is an important omission, considering that patient safety is a relatively new field and many senior physicians have not received specific training in this area [47]. Ahmed et al. [48] proposed that educational interventions should be undertaken to create awareness of the complexity of patient safety and pointed out that students are an underexploited resource for identifying safety problems and proposing solutions for them.

Finally, the essential challenge is to determine whether the professionals participating in the intervention have modified their behavior with respect to patient safety to provide better care, and whether patients’ outcomes did improve.

## Conclusions

The current study indicates the effectiveness of the educational intervention given to residents and their tutors in family medicine teaching units with regard to patient safety culture measured by the number of events reported. In the future, it would be interesting to replicate this study in teaching units in other Health Departments from different countries and including other professionals in addition to residents in family and community medicine.

The significant association observed among organizational dimensions and SOPS outcomes evidences their impact on patient safety culture and shows the path for implementation of changes with a complete system view.

## Additional files

Additional file 1: Hospital Survey on Patient Safety Culture: Items and Dimensions. (DOCX 12 kb)

Additional file 2: The TIDieR (Template for Intervention Description and Replication) Check. (DOCX 12 kb)

Additional file 3: Basal organizational variables by group. (DOCX 15 kb)

Additional file 4: Summary results of the intervention for each outcome variable, crude and adjusted models (intention-to-treat). (DOCX 11 kb)

## Abbrevations

AIC: Akaike Information Criterion
MCAR: Missing Completely At Random
MI: Multiple Imputation
Ref: Reference
RRR: Relative Risk Reduction
SOPS: Survey On Patient Safety culture.
