# Supplementary material for: Effectiveness of an educational intervention to improve the safety culture in primary care: a randomized trial
Source: BMC Fam Pract. 2019 Jan 18;20:15. doi: 10.1186/s12875-018-0901-8 (PMC6337818; doi:10.1186/s12875-018-0901-8)
Supplement: Supplementary file 1 — Hospital Survey on Patient Safety Culture: Items and Dimensions. (DOCX 12 kb) [file 12875_2018_901_MOESM1_ESM.docx]

| **Additional file 1. Hospital Survey on Patient Safety Culture: Items and Dimensions (18)** |
| --- |
| **1. Teamwork Within Units**  (Strongly Disagree, Disagree, Neither Agree nor Disagree, Agree, Strongly Agree)  . People support one another in this unit.  . When a lot of work needs to be done quickly, we work together as a team to get the work done.  . In this unit, people treat each other with respect.  . When one area in this unit gets really busy, others help out.  **2. Supervisor/Manager Expectations & Actions Promoting Patient Safety**  (Strongly Disagree, Disagree, Neither Agree nor Disagree, Agree, Strongly Agree)  . My supervisor/manager says a good word when he/she sees a job done according to established patient safety procedures.  . My supervisor/manager seriously considers staff suggestions for improving patient safety.  . Whenever pressure builds up, my supervisor/manager wants us to work faster, even if it means taking shortcuts. (negatively worded)  . My supervisor/manager overlooks patient safety problems that happen over and over. (negatively worded)    **3. Organizational Learning—Continuous Improvement**  (Strongly Disagree, Disagree, Neither Agree nor Disagree, Agree, Strongly Agree)  . We are actively doing things to improve patient safety.  . Mistakes have led to positive changes here.  . After we make changes to improve patient safety, we evaluate their effectiveness.  **4. Management Support for Patient Safety**  (Strongly Disagree, Disagree, Neither Agree nor Disagree, Agree, Strongly Agree)  . Hospital management provides a work climate that promotes patient safety.  . The actions of hospital management show that patient safety is a top priority.  . Hospital management seems interested in patient safety only after an adverse event happens. (negatively worded)  **5. Overall Perceptions of Patient Safety**  (Strongly Disagree, Disagree, Neither Agree nor Disagree, Agree, Strongly Agree)  . Patient safety is never sacrificed to get more work done.  . Our procedures and systems are good at preventing errors from happening.  . It is just by chance that more serious mistakes don't happen around here. (negatively worded)  . We have patient safety problems in this unit. (negatively worded)  **6. Feedback & Communication About Error**  (Never, Rarely, Sometimes, Most of the time, Always)  . We are given feedback about changes put into place based on event reports.  . We are informed about errors that happen in this unit.  . In this unit, we discuss ways to prevent errors from happening again.  **7. Communication Openness**  (Never, Rarely, Sometimes, Most of the time, Always)  . Staff will freely speak up if they see something that may negatively affect patient care.  . Staff feel free to question the decisions or actions of those with more authority.  . Staff are afraid to ask questions when something does not seem right. (negatively worded)  **8. Frequency of Events Reported**  (Never, Rarely, Sometimes, Most of the time, Always)  . When a mistake is made, but is caught and corrected before affecting the patient, how often is this reported?  . When a mistake is made, but has no potential to harm the patient, how often is this reported?  . When a mistake is made that could harm the patient, but does not, how often is this reported?  **9. Teamwork Across Units**  (Strongly Disagree, Disagree, Neither Agree nor Disagree, Agree, Strongly Agree)  . There is good cooperation among hospital units that need to work together.  . Hospital units work well together to provide the best care for patients.  . Hospital units do not coordinate well with each other. (negatively worded)  . It is often unpleasant to work with staff from other hospital units. (negatively worded)  **10. Staffing**  (Strongly Disagree, Disagree, Neither Agree nor Disagree, Agree, Strongly Agree)  . We have enough staff to handle the workload.  . Staff in this unit work longer hours than is best for patient care. (negatively worded)  . We use more agency/temporary staff than is best for patient care. (negatively worded)  . We work in "crisis mode" trying to do too much, too quickly. (negatively worded)  **11. Handoffs & Transitions**  (Strongly Disagree, Disagree, Neither Agree nor Disagree, Agree, Strongly Agree)  . Things "fall between the cracks" when transferring patients from one unit to another.  (negatively worded)  . Important patient care information is often lost during shift changes. (negatively worded)  . Problems often occur in the exchange of information across hospital units. (negatively worded)  . Shift changes are problematic for patients in this hospital. (negatively worded)  **12. Nonpunitive Response to Errors**  (Strongly Disagree, Disagree, Neither Agree nor Disagree, Agree, Strongly Agree)  . Staff feel like their mistakes are held against them. (negatively worded)  . When an event is reported, it feels like the person is being written up, not the problem.  (negatively worded)  . Staff worry that mistakes they make are kept in their personnel file. (negatively worded)  **Patient Safety Grade**  (Excellent, Very Good, Acceptable, Poor, Failing)  . Please give your work area/unit in this hospital an overall grade on patient safety.  **Number of Events Reported**  (No event reports, 1 to 2 event reports, 3 to 5 event report, 6 to 10 event reports, 11 to 20 event reports, 21 event reports or more)  . In the past 12 months, how many event reports have you filled out and submitted. |
