# Supplementary material for: Effectiveness of an educational intervention to improve the safety culture in primary care: a randomized trial
Source: BMC Fam Pract. 2019 Jan 18;20:15. doi: 10.1186/s12875-018-0901-8 (PMC6337818; doi:10.1186/s12875-018-0901-8)
Supplement: Supplementary file 3 — Basal organizational variables by group. (DOCX 15 kb) [file 12875_2018_901_MOESM3_ESM.docx]

**Additional file 3. Basal organization variables by groups^.^**

|  |  | **Intervention (N=53)** | | | **Control (N=46)** | | |
| --- | --- | --- | --- | --- | --- | --- | --- |
|  | | n | (%) | n | | | (%) |
| Health area | |  |  |  | | |  |
|  | A Coruña-Ferrol | 13 | (24.50) | 10 | | (21.70) | |
|  | Lugo | 5 | (9.40) | 6 | | (13.00) | |
|  | Ourense | 10 | (18.90) | 7 | | (15.20) | |
|  | Pontevedra | 5 | (9.40) | 6 | | (13.00) | |
|  | Santiago | 8 | (15.10) | 3 | | (6.50) | |
|  | Vigo | 12 | (22.60) | 14 | | (30.40) | |
| Tenure with Health Center | |  |  |  | | |  |
|  | Less than 1 year | 1 | (1.90) | 2 | | (4.30) | |
|  | 1 to 5 years | 6 | (11.30) | 5 | | (10.90) | |
|  | 6 to 10 years | 1 | (1.90) | 1 | | (2.20) | |
|  | 11 to 15 years | 7 | (13.20) | 4 | | (8.70) | |
|  | 6 to 20 years | 9 | (17.00) | 5 | | (10.90) | |
|  | 21 years or more | 3 | (5.70) | 6 | | (13.00) | |
|  | Resident | 26 | (49.10) | 23 | | (50.00) | |
| Tenure in Profession | |  |  |  | | |  |
|  | 6 to 10 years | 2 | (3.80) | 1 | | (2.20) | |
|  | 11 to 15 years | 3 | (5.70) | 2 | | (6.50) | |
|  | 16 to 20 years | 12 | (22.60) | 5 | | (10.90) | |
|  | 21 years or more | 7 | (18.90) | 12 | | (30.40) | |
|  | Resident | 21 | (49.10) | 16 | | (50.00) | |
| Hours Worked per Week | |  |  |  | | |  |
|  | Less than 20 hours/week | 1 | (1.90) | 0 | | (0.00) | |
|  | 20 to 39 hours/week | 34 | (65.40) | 31 | | (67.40) | |
|  | 40 to 59 hours/week | 16 | (30.80) | 13 | | (28.30) | |
|  | 60 hours/week or more |  | (1.90) |  | | (4.30) | |
| Direct Patient Contact | |  |  |  | | |  |
|  | No | 0 | (0.00) | 0 | | (0.00) | |
|  | Yes | 53 | (1.00) | 46 | | (100.00) | |
