# Supplementary material for: Effectiveness of an educational intervention to improve the safety culture in primary care: a randomized trial
Source: BMC Fam Pract. 2019 Jan 18;20:15. doi: 10.1186/s12875-018-0901-8 (PMC6337818; doi:10.1186/s12875-018-0901-8)
Supplement: Supplementary file 4 — Summary results of the intervention for each outcome variable, crude and adjusted models (intention-to-treat). (DOCX 11 kb) [file 12875_2018_901_MOESM4_ESM.docx]

**Additional file 4. Summary results of the intervention for each outcome variable, crude and adjusted model (intention-to-treat).^§^**

| **Patient safety grade. Generalized lineal model, family gaussian (identity link).** | | | | | | | | | |
| --- | --- | --- | --- | --- | --- | --- | --- | --- | --- |
|  | **Crude model** |  |  |  |  | **Adjusted model** |  |  |  |
| Intercept | 2.91*** | (0.12) |  |  |  | 1.16*** | (0.23) |  |  |
| Intervention group (control group=ref) | 0.00 | (0.16) |  |  |  | -0.03 | (0.13) |  |  |
| R1. Patient safety grade (basal) |  |  |  |  |  | 0.62*** | (0.10) |  |  |
|  |  |  |  |  |  |  |  |  |  |
| **Number of events reported. Multinomial model.** | | | | | | | | | |
|  | **Crude model** |  |  |  |  | **Adjusted model** |  |  |  |
|  | 1 to 2 (none = ref) |  | 3 or more (none = ref) |  |  | 1 to 2 (none = ref) |  | 3 or more (none = ref) |  |
| Intercept | -0.77 | (0.35) | -2.70 | (0.73) |  | 2.78 | (30.64) | -0.65 | (104.38) |
| Intervention group (control group=ref) | -0.08 | (0.50) | 2.26*** | (0.80) |  | 0.17 | (0.61) | 2.52*** | (0.89) |
| N. events reported (basal) 1 to 2 |  |  |  |  |  | 7.04 | (64.99) | 3.80 | (221.43) |
| N. events reported (basal) 3 or more |  |  |  |  |  | 1.34 | (37.53) | -0.34 | (127.84) |
| ^§^ Data as Estimate (SD) | |  |  |  |  |  |  |  |  |
| *** p < 0.001 |  |  |  |  |  |  |  |  |  |
